# The Canary in the Coal Mine Tweets: Social Media Reveals Public Perceptions of Non-Medical Use of Opioids

**DOI:** 10.1371/journal.pone.0135072

**Published:** 2015-08-07

**Authors:** Brian Chan, Andrea Lopez, Urmimala Sarkar

**Affiliations:** Division of General Internal Medicine, School of Medicine, University of California San Francisco, San Francisco, California, United States of America; Illinois Institute of Technology, UNITED STATES

## Abstract

**Objective:**

Non-medical prescription opioid use is a growing public health concern. Social media is an emerging tool to understand health attitudes, beliefs, and behaviors.

**Methods:**

We retrieved a sample of publicly available Twitter messages in early 2014, using common opioid medication names and slang search terms. We used content analysis to code messages by user, context of message (personal vs general experiences), and key content themes.

**Results:**

We reviewed 540 messages, of which 375 (69%) messages were related to opioid behaviors. Of these, 316 (84%) originated from individual user accounts; 125 messages expressed personal experience with opioids. The majority of personal messages referenced using opioids to obtain a “high”, use for sleep, or other non-intended use (87,70%). General attitudes regarding opioid use included positive sentiment (52, 27%), comments on others peoples opioid use (57, 30%), and messages containing public health information or links (48, 25%).

**Conclusions:**

In a sample of social media messages mentioning opioid medications, the most common theme amongst English users related to various forms of opioid misuse. Social media can provide insights into the types of misuse of opioids that might aid public health efforts to reduce non-medical opioid use.

## Introduction

Non-medical prescription opioid use is a rising public health concern. Prescriptions for opioid medications have nearly doubled between 1994 and 2007.[1] The burden of illness is high; opioid prescription related deaths (14,800) made up 73.8% of the 20,044 prescriptions drug overdose deaths in 2008.[2] The prevalence of patients who report a non-physician source of opioid medicine is reported to be more than double (69%) those who report a physician source (31%) in a recent analysis of the National Survey on Drug Use and Health.[3] With the concern over the growing epidemic of non-medical use of prescription opioids,[4, 5] there is a need to understand the changing public attitudes and beliefs about these medications. The traditional methods of characterizing non-medical use, such as large government-funded telephone surveys, carry limitations including underreporting, and a significant delay between data collection and public data availability.[6, 7]

Social media is a means to enhance real-time understanding of attitudes and beliefs regarding opioid use, because it provides an opportunity to share ideas, opinions, and information instantaneously and publicly online. The micro-blogging site Twitter has experienced rapid growth over time with an estimated 18% of US online adults using Twitter in 2013, and about half of those reporting daily use of the application.[8] Twitter permits communication with real-life social networks to be both public and online; therefore, personal information and potentially stigmatizing behaviors that individuals would previously share only with close contacts, such as substance abuse,[9] are often available for research and to inform public health efforts and policy.

We conducted an exploratory, mixed-methods analysis of Twitter messages to characterize the nature of the content relating to opioid medications. In this study, we aimed to describe who uses social media to discuss opioid use or misuse, and what attitudes, themes, and behaviors social media users message about. Our hypothesis was that the anonymity of social media allowed for more candid discussion of opioid misuse and abuse behaviors.

## Methods

### Study Setting: Twitter

**Fig 1:**
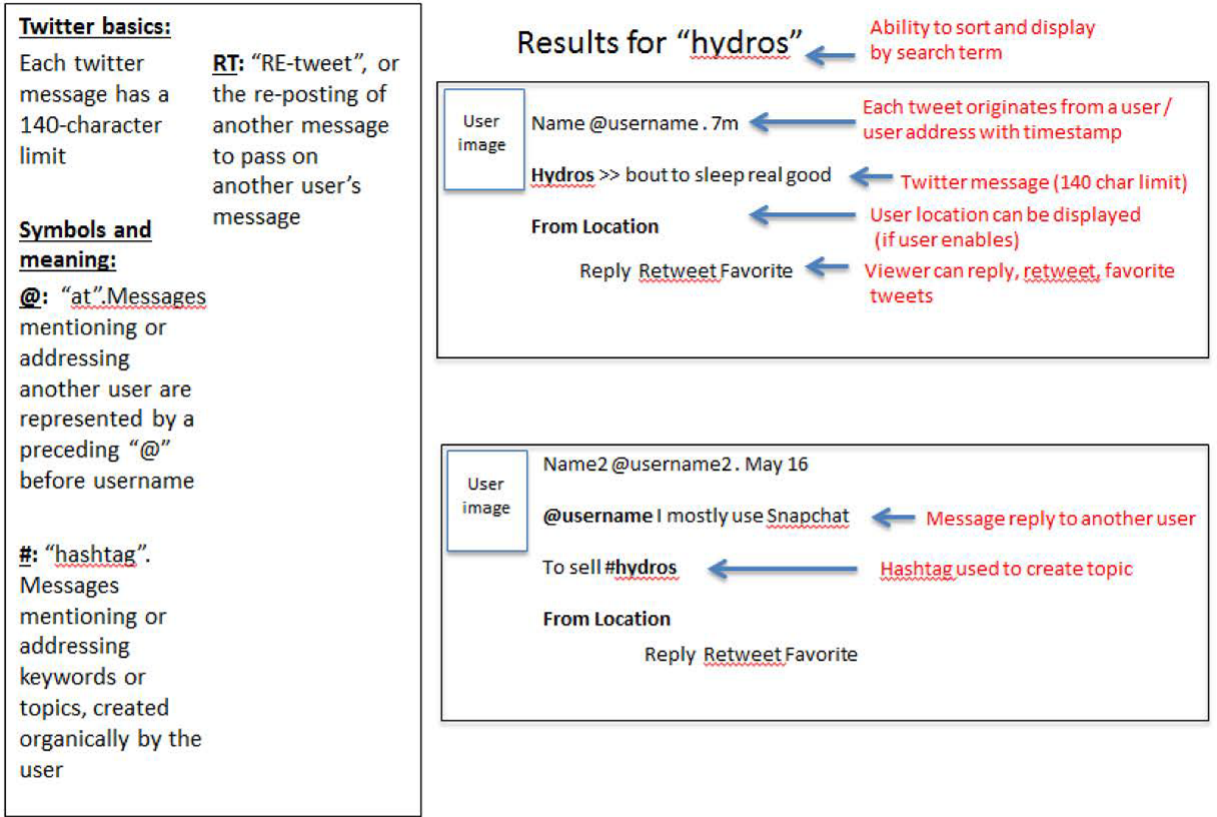
An overview of Twitter: re-created example using search term “hydros”.

Twitter (www.twitter.com) is an online social networking site that allows individuals to share information in short messages called “tweets” that are 140 characters or less. Twitter is largely a public forum, and accounts can be individual (from friends and family to celebrities and politicians) or organizational (including national and local non-profit organizations, companies, advocacy groups, and others) to media outlets posting real-time updates. Users form personalized networks by following the “feed” (message stream) of other user accounts, creating a timeline of personalized news and information (Fig 1). Similarly, users gain influence by having “followers” who receive their tweets. Although the text of each message is limited, the content shared is often rich, especially through links to longer stories, entire websites, pictures, and/or videos.

### Data Collection

We evaluated the content of a cross-sectional sample of publicly available Twitter messages during a 2 week period in March and early April 2014. We did not query Twitter’s API, but instead manually searched Twitter messages using the following search terms: 1) Duragesic, 2) Fentanyl 3) Hydrocodone, 4) Hydros, 5) Oxy, 6) Oxycodone, 7) Oxycotin, 8) Oxycotton, 9) Vicodin, 10) Vikes, and 11) Oxycontin. We identified search terms based on prior literature on the most commonly prescribed opioids, using generic, trade, and slang terms.[10] We conducted searches during different days of the week and times of day to sample across potential time dependent variations in themes, similar to previous studies using Twitter.[11–13] “We used a consecutive sampling scheme to compile Twitter messages up to a maximum of 50 per search term. We used a 3^rd^ party web-available program, tweetseeker.com (http://www.tweetseeker.com) to export our searches into Excel.

### Qualitative Coding

We initially used content analysis[14, 15] to code each Twitter message. First, we developed codes deductively using commonly accepted definitions of prescription drug abuse[16] and aberrancy behaviors,[17] an existing opioid misuse tool, the Current Opioid Misuse Measure (COMM)[18], as well as a prior study of Twitter regarding Adderall use amongst college students.[19] We also developed codes based on prior qualitative studies of social media messages, including attitudes regarding breast and cervical cancer screening,[20] physician office experiences,[12] and tobacco product use.[21]

**Fig 2:**
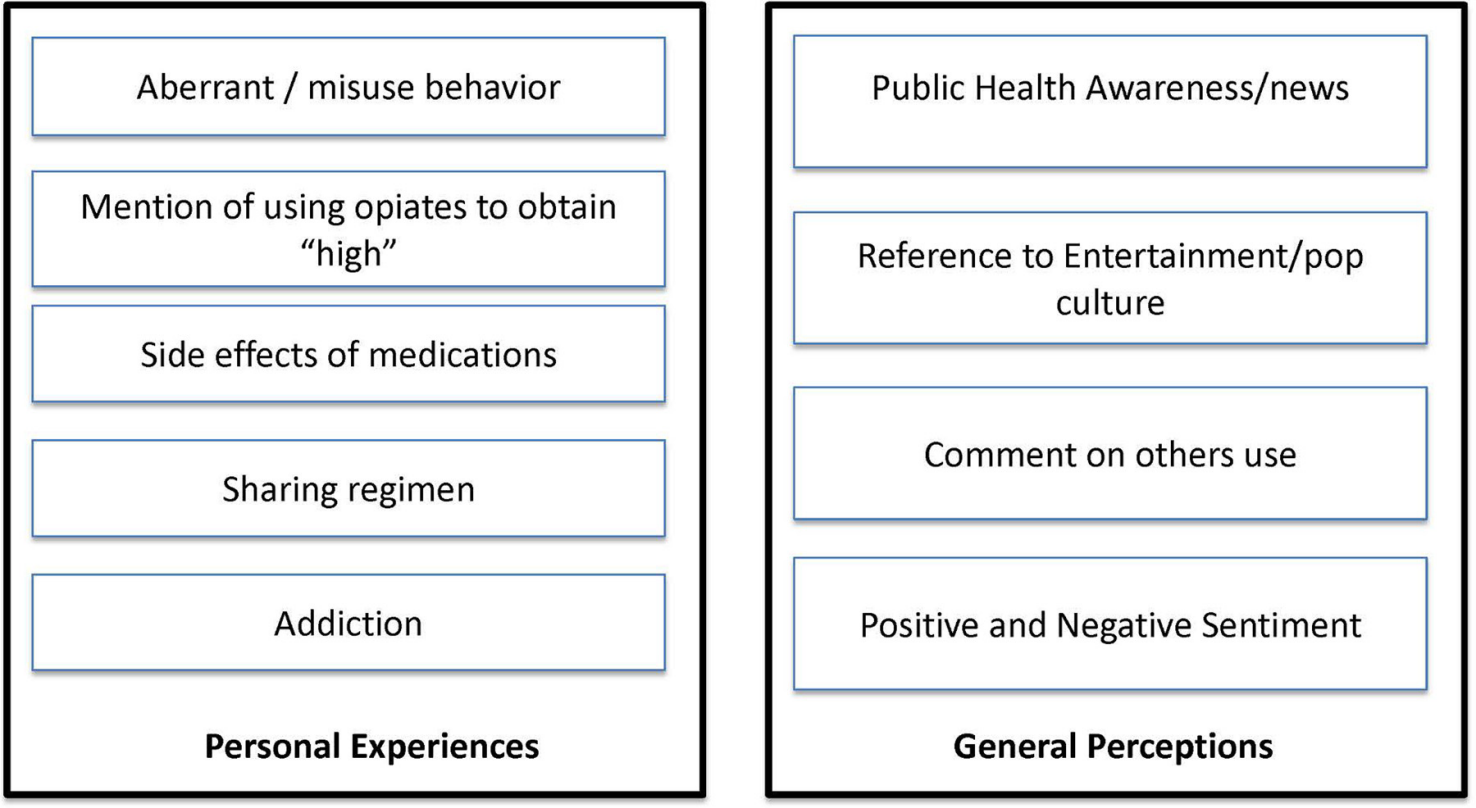
Conceptual Framework for Categorizing Twitter Messages Containing Personal Experiences and General Perceptions.

Next, we developed codes inductively, through a grounded theory approach, as additional themes arose not captured by existing thematic coding schemes.[22] Because we were interested in user attitudes and behaviors, we focused on the themes derived from individual user messages. First, one member of the team (BC) examined a 20% sample of all messages to develop a coding framework that included five major themes for personal messages (those messages that reflected personal experience with opioid medication), and four major themes in general messages containing opioid search terms (Fig 2). Next, the research team (AL, BC, US) examined an additional 30% of all tweets and met to refine the overall coding framework that included author type, message category (personal versus general), and description of reported behavior or perception. For example, we coded the message “This Liquid Oxycodone Got me Leaning (smiling emoticon)” as from an individual author, blogging a personal statement about use, with content of the tweet relating to using medication to obtain a high. We resolved discrepancies by regular meetings and discussion and revised the codebook iteratively. Data were compiled in an Excel spreadsheet to directly compare categorization of tweets across coders. If new categories (including sub-categories) emerged, we changed the coding framework and re-analyzed the messages according to the new structure. Once no new categories or themes emerged, reaching thematic saturation, the coding scheme was finalized and one member of the team coded the remaining messages (AL), followed by a joint review of aggregated messages. Because we included a substantial number of messages in our sample, we report frequencies for our data below.

## Results

Overall, we searched a total of 540 Twitter messages. Of these, we excluded 182 (33%) messages for the following reasons: non-English content (20, 4%), content referring to non-opioid use of search term (138, 26%), and inability to discern the context of the message in a meaningful manner (24, 4%). For example, 48 of 50 messages using the search term “Vikes”, a slang term for Vicodin, contained content relating to the Minnesota Vikings football team. We also excluded messages originating from users names that contained search terms but whose messages did not. We coded the remaining 375 messages.

**Fig 3:**
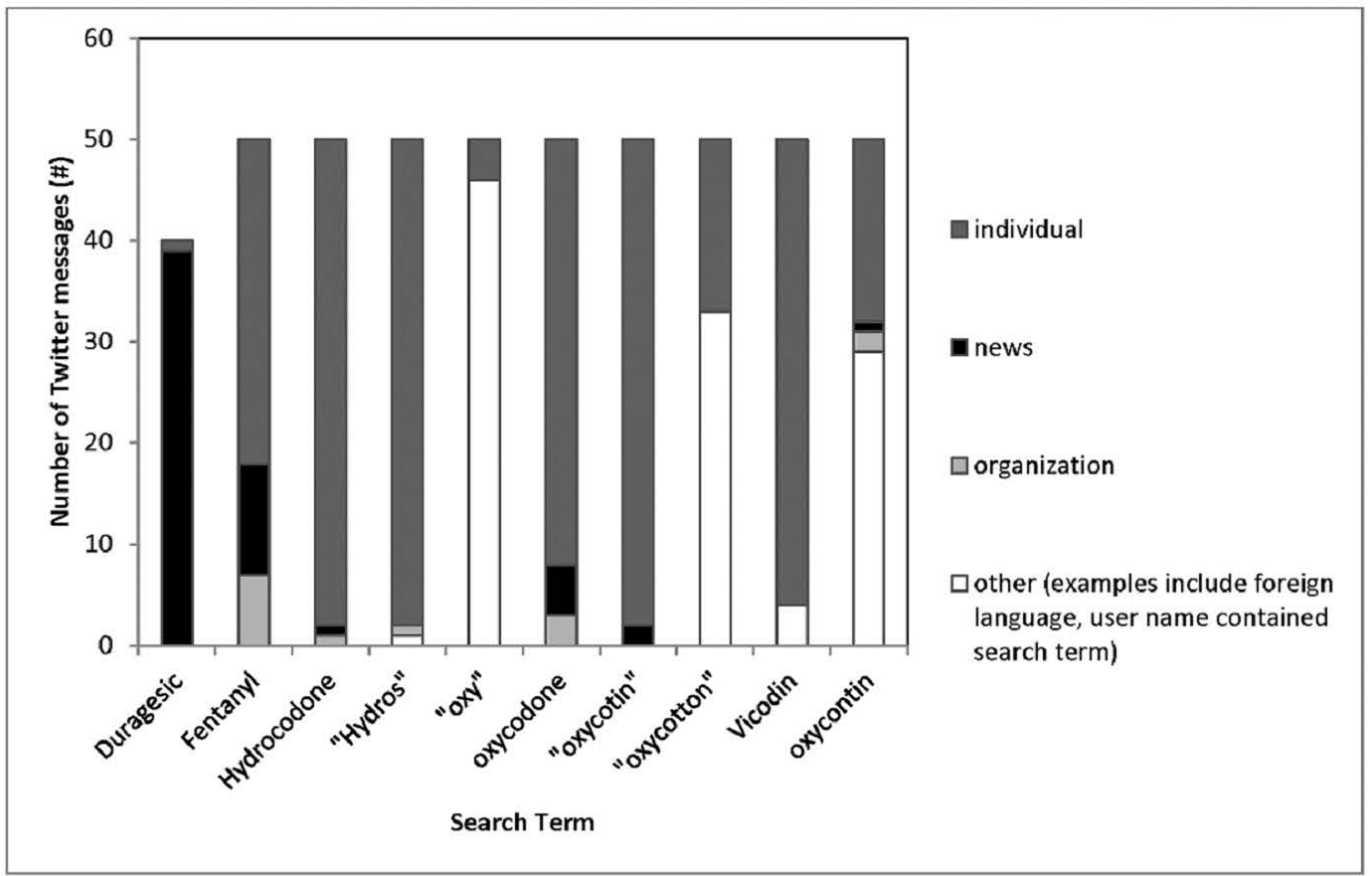
Author of Twitter Messages (individuals, organizations, news outlets, other (user name contained search terms, foreign languages, references to non-opioids).

Of the 375 messages, 333 were from unique user accounts (89%). Individual accounts made up the majority of authored messages (316, 84%), compared to organizations or news outlet sources (59, 16%). The distribution of author type varied depending on the search term (Fig 3). The majority of messages authored by individuals were general comments on opioid use (191, 60%), and included 65 “retweets,” messages that are seen by a Twitter users “followers” and subsequently re-sent on their own network. There were 125 (40%) messages reflecting personal experience.

### Personal experiences with opioid use by Twitter users

The majority of Twitter messages of a personal nature contained references to aberrant and opioid misuse behaviors (Table 1). Of the 125 personal messages, 87 (70%) contained themes of opioid prescription misuse, or aberrant behaviors Twitter users were candid in messaging about using opioids to obtain a “high” (26, 21%). An example is a user who messaged “Bought some **oxycontin**, we finna get trippy mane” (sic). Co-use with other substances was explicitly mentioned in 16 (13%) of personal messages, (eg. “I’m eating shrooms, popping **oxycontin**”). A similar number of messages contained themes of seeking to obtain opioid medication (16, 13%).

Other personal themes included sharing one’s pain regimen with other followers without mention of aberrant or misuse behavior (26, 21%). For example, one user messaged “On more antibiotics, ibuprofen, and **vicodin**, with a scheduled wisdom tooth extraction in May. yay!” An equal number of messages contained mention of side effects of opioid medications (16, 13%), with a representative message “…I usually skip the **hydrocodone** because that’s insta-barf medicine for me.” A small percentage of personal messages referenced addiction to opioid medications (5, 4%).

**Table 1:** Major opioid related content and attitudes contained in Personal Twitter messages, with representative quotes and frequencies. (n = 125).

Theme:	Representative Message:	Frequency # (%):
**Aberrant/misuse behaviors**		
Use of opioids for sleep*	2 hydros and back to sleep (;	11 (9%)
Co-Use* ^a^	Vicodin and vodka get me in my drake like a champ	16 (13%)
	2 hydros, 40oz, and Mary	
Seeking*	Anyone know where I can get some Hydrocodone's? Hmu	16 (13%)
Selling	Don’t know if I should sell all these hydrocodone pills hmmm	2 (2%)
Improper Administration*	You need to not brag about popping vicodin/oxy if you dont even know how to cold water extract the good shit away from the fillers and APAP	3 (2%)
Using other’s medicine *	Thanks. Co-worker slipped me something and, hot damn, it seems to be working. May be Allegra. May be Vicodin. Either way: D	2 (2%)
Poor Response To Medication	@XXX I'm on Vicodin but it doesn't do shit but make me sleep lol	11 (9%)
**Mention of using opiates to obtain “high”** *	reoccurring hydrocodone dreams that i'm too fucked up to walk forward and i keep falling backwards and spinning and shit	26 (21%)
**Side Effects** ^**a**^	I took one of my (prescribed) hydros once and fought the sleep cause i had shit to do…wasnt fun at allDruggies are weird	16 (13%)
**Sharing personal regimen**	How I get just slightly enough pain away to not be in bed all day like I'm depressed: 45mgs Oxycodone, 6 Advils, high grade MMJ.	26 (21%)
**Addiction**	Ever since I stuck Fentanyl patches on and took tramset (I fink) tablets I been addicted 2 painkillers lol	5 (4%)

* Coding theme based on the Current Opioid Misuse Measure (COMM)

^a^ Coding theme based on prior study of Twitter and Adderall themes

### General Perceptions of opioid use by Twitter users

Many messages from personal accounts (191, 60%) did not refer to personal experiences but to more general perceptions of opioid use (Table 2). Of these messages, we noted 52 (27%) as expressing a positive sentiment toward opioids (eg. “**Hydros** fix everything”), compared to 15 (8%) of messages expressing a negative sentiment toward opioid use (eg. “I don’t understand the culture of young people who think its okay to pop Molly’s, Percocet, **Oxycotin**, Xanax, etc.”) A number of Twitter messages contained commentary on inappropriate use of opioids by others in their social circle (57, 30%). There were 30 (16%) messages that referenced opioid use in entertainment media, music lyrics containing opioid use, or public figures. A number of messages from individuals touched on public health awareness or posted links to media stories on opioid related awareness (eg. “Crazy documentary on the grip prescription drugs have on our society. topdocumentaryfilms.com/**oxycontin**-expr…”)

**Table 2:** Major opioid related content and attitudes contained in General Twitter messages, with representative quotes and frequencies. (n = 191 includes 65 re-tweets).

Theme:	Representative Message:	Frequency # (%):
**Public Health Awareness Statement**	Before you reach for a Hydrocodone, read this: bit.ly/1fiNVuO	48 (25%)
**Reference to Entertainment (Music, Rap, TV, Movies)**	Popping Vicodin like my names Dr. House	30 (16%)
**Negative Sentiment toward Opioid**	I don’t understand the culture of young people who think it’s okay to pop Molly’s, Percocet, Oxycotin, Xanax, etc.	15 (8%)
**Positive Sentiment toward Opioid**	@XXX @XXX hydros fix everything	52 (27%)
**Comment on other’s use**	My psyc. Teacher just said the cure for anything is a hydrocodone and a shot of whiskey	57 (30%)
**Medical Setting**	RT @XXXmd: @XXXmd I don’t have a document, but in our ED, almost everyone tubed gets ASAP a midazolam and fentanyl drip	19 (10%)
**Retweet of other’s message or link**	RT @XXXNews: Addicts abusing Fentanyl don’t realize how their addiction affects their family. fentanylabuse.org 800-303-2482 httpic.twitter.com/QRt1jMWl1E	65 (34%)
**Others**		
- question to others	What u gata do to be on oxycotton?	2 (1%)
- unclear context	@o_XXX #oxycotton	5 (3%)

### Inclusion of Slang Terms, Misspelled Search Terms

Of 123 messages that included slang or misspelled terms, 55 messages were coded as containing reference to misuse/abuse behavior or attitudes (45%). This is compared to 53 of 252 (21%) of messages that included trade or generic names of opioids.

## Discussion

In this qualitative analysis of self-reported attitudes and behaviors about opioids online, we found that Twitter users publicly message both personal experience with opioid medications, and general perceptions regarding opioid medications, including content related to non-medical use of opioid medications. While Twitter messages should not replace traditional survey methods of opioid use, the content contained in social media messages can be a useful, real-time, publicly-available source of information to be used in conjunction to identify emerging trends in use and misuse behavior, as well as a forum to communicate public health awareness on non-medical use of opioids.

The personal experience messages captured a wide variety of opioid related behaviors and are consistent with reported behaviors found in other survey methods about misuse and abuse of opioid medications.[3] Authors messaged about their aberrant use of opioids as a sleep or anti-anxiolytic, or as a way to obtain a high sensation. Similarly, authors shared openly messages about co-use of opioids with other medications, illegal drugs, or alcohol. Although we cannot quantify the magnitude of positive opioid-related sentiment based on our manual sample, the high frequency of messages that display aberrant opioid behaviors in a positive light are deeply concerning, and suggest that the public still lacks sufficient awareness of the mounting harm associated with opioid misuse. Twitter may be useful in the future for tracking public awareness of opioid misuse.

These messages also provide data for public health practitioners in real-time, providing data that is complementary to traditional methods. Characterizing risky behaviors through health surveys or adverse events is subject to limitations in response rates and significant time delays.[7] In-clinic patient reported screening tools are limited by recall bias, social-desirability bias, and concerns about receiving medication.[23, 24] The real-time nature of Twitter messaging, as well as the relative anonymity of Twitter, can complement data from current surveys and reporting systems.

Our findings are similar to studies that used Twitter to characterize potential misuse of other prescribed medications, like the use of Adderall as a study aid.[19] Prior studies report Twitter as a means for studying behavior with other health related behaviors such as smoking and drinking alcohol.[21, 25] These prior studies have all used automated algorithms to study text data from twitter, while we used traditional qualitative analysis, which permitted some additional insight into each message that automated “big data” analyses cannot provide. As an example, the initial automated search terms in our sample did not result in a precise identification of opioid-related messages, and we restricted our analysis to pertinent messages. There is promise in combining traditional qualitative analysis with automated approaches, such an approach led to greater precision in a study of online doctor ratings.[26]

Our results reveal several lessons in future use of social media data sources for opioid misuse surveillance. First, themes related to personal experience with opioids differ from themes related to general perceptions of opioids, the latter which is influenced by one’s social network, pop culture, news, and entertainment sources. If actual behaviors are to be studied, future search algorithms will need to differentiate personal from general experiences. Second, searching of slang terms (“oxy”) or misspelled opioid search terms (“oxycotin”) yielded informative messages that referenced opioid use, misuse, and abuse. Design of social media surveillance programs need to consider how to include misspellings or slang terms into their algorithms to improve effectiveness.”

In addition to potential use as a surveillance tool, we also found that Twitter can be a source of information for the public. Organizations tended to message about the potential risks and harms of opioid medications, or retweeted new stories about criminal activity involving opioid medications. Prior use of twitter during political movements underscores the immediacy of the medium,[27] and we believe this is an advantage for public health applications as well.

### Limitations

This exploratory analysis has several limitations. By necessity, a manual qualitative analysis has a limited sample size and scope. We cannot speak to the representativeness of the messages we sampled, though we did attempt to include different times of day, days of the week, as well as scientific, trade, and slang terms. We pre-specified the search terms based on a preliminary review of social media related to prescription opioid use. These terms were subject to “noise” from other non-opioid related messages. Future work should examine additional keywords and messages over a longer period of time to target specific behavior or content. We acknowledge that while Twitter use amongst adults is growing, people who message about opioid use are different from the general population, and our findings should be interpreted in that light. However, a recent Twitter based surveillance study have produced similar findings to traditional survey designs in characterizing Adderall abuse, [19] and we believe the advantages in cost and immediacy of data availability confer significant advantages over traditional methods to study high-risk behaviors.

## Conclusion

Social media has changed the way individuals communicate about health-related behaviors, including behaviors that are illicit and difficult to capture otherwise. A next step is combining results from a qualitative analysis with larger automated data in order to develop search strategies to monitor opioid use and misuse behaviors that can be compared to gold standard surveillance systems currently in place. In addition, user location information provided on messages may offer additional information to identify incipient public health concerns. Twitter has potential to complement existing public health approaches to characterize opioid medication misuse and to develop effective public health campaigns to address this growing epidemic.

## References

[1] Volkow ND, McLellan TA, Cotto JH, Karithanom M, Weiss SR. Characteristics of opioid prescriptions in 2009. JAMA: the journal of the American Medical Association. 2011;305(13):1299–301. Epub 2011/04/07. 10.1001/jama.2011.401 21467282 PMC3187622

[2] Centers for Disease Control, Prevention. Vital signs: overdoses of prescription opioid pain relievers---United States, 1999–2008. MMWR Morbidity and mortality weekly report. 2011;60(43):1487–92. Epub 2011/11/04. . 22048730

[3] Becker WC, Tobin DG, Fiellin DA. Nonmedical use of opioid analgesics obtained directly from physicians: prevalence and correlates. Archives of internal medicine. 2011;171(11):1034–6. Epub 2011/06/15. 10.1001/archinternmed.2011.217 . 21670373

[4] Volkow ND, Frieden TR, Hyde PS, Cha SS. Medication-assisted therapies—tackling the opioid-overdose epidemic. The New England journal of medicine. 2014;370(22):2063–6. 10.1056/NEJMp1402780 . 24758595

[5] Alexander GC, Kruszewski SP, Webster DW. Rethinking opioid prescribing to protect patient safety and public health. JAMA: the journal of the American Medical Association. 2012;308(18):1865–6. Epub 2012/11/15. 10.1001/jama.2012.14282 . 23150006

[6] Freifeld CC, Brownstein JS, Menone CM, Bao W, Filice R, Kass-Hout T, et al Digital drug safety surveillance: monitoring pharmaceutical products in twitter. Drug safety: an international journal of medical toxicology and drug experience. 2014;37(5):343–50. Epub 2014/04/30. 10.1007/s40264-014-0155-x 24777653 PMC4013443

[7] Roehr B. US hospital incident reporting systems do not capture most adverse events. Bmj. 2012;344:e386 10.1136/bmj.e386 . 22246454

[8] Duggan M SA. Social Media Update 2013. Pew Research Internet Project. 2013.

[9] Moreno MA, Egan KG, Brockman L. Development of a researcher codebook for use in evaluating social networking site profiles. The Journal of adolescent health: official publication of the Society for Adolescent Medicine. 2011;49(1):29–35. Epub 2011/06/28. 10.1016/j.jadohealth.2011.04.015 21700153 PMC3125574

[10] Manchikanti L, Helm S 2nd, Fellows B, Janata JW, Pampati V, Grider JS, et al Opioid epidemic in the United States. Pain physician. 2012;15(3 Suppl):ES9–38. Epub 2012/07/20. . 22786464

[11] Lagu T, Hannon NS, Rothberg MB, Lindenauer PK. Patients' evaluations of health care providers in the era of social networking: an analysis of physician-rating websites. Journal of general internal medicine. 2010;25(9):942–6. 10.1007/s11606-010-1383-0 20464523 PMC2917672

[12] Lopez A, Detz A, Ratanawongsa N, Sarkar U. What patients say about their doctors online: a qualitative content analysis. Journal of general internal medicine. 2012;27(6):685–92. 10.1007/s11606-011-1958-4 22215270 PMC3358396

[13] Kim AE, Hansen HM, Murphy J, Richards AK, Duke J, Allen JA. Methodological considerations in analyzing twitter data. Journal of the National Cancer Institute Monographs. 2013;2013(47):140–6. Epub 2014/01/08. 10.1093/jncimonographs/lgt026 . 24395983

[14] Borzekowski DL, Schenk S, Wilson JL, Peebles R. e-Ana and e-Mia: A content analysis of pro-eating disorder Web sites. Am J Public Health. 2010;100(8):1526–34. Epub 2010/06/19. 10.2105/AJPH.2009.172700 20558807 PMC2901299

[15] Jenssen BP, Klein JD, Salazar LF, Daluga NA, DiClemente RJ. Exposure to tobacco on the internet: content analysis of adolescents' internet use. Pediatrics. 2009;124(2):e180–6. Epub 2009/07/22. 10.1542/peds.2008-3838 19620193 PMC2818533

[16] Chabal C, Erjavec MK, Jacobson L, Mariano A, Chaney E. Prescription opiate abuse in chronic pain patients: clinical criteria, incidence, and predictors. The Clinical journal of pain. 1997;13(2):150–5. . 9186022 10.1097/00002508-199706000-00009

[17] Wachholtz A, Gonzalez G, Boyer E, Naqvi ZN, Rosenbaum C, Ziedonis D. Intersection of chronic pain treatment and opioid analgesic misuse: causes, treatments, and policy strategies. Substance abuse and rehabilitation. 2011;2:145–62. 10.2147/SAR.S12944 24474854 PMC3846312

[18] Butler SF, Budman SH, Fernandez KC, Houle B, Benoit C, Katz N, et al Development and validation of the Current Opioid Misuse Measure. Pain. 2007;130(1–2):144–56. Epub 2007/05/12. 10.1016/j.pain.2007.01.014 17493754 PMC1950245

[19] Hanson CL, Burton SH, Giraud-Carrier C, West JH, Barnes MD, Hansen B. Tweaking and tweeting: exploring Twitter for nonmedical use of a psychostimulant drug (Adderall) among college students. Journal of medical Internet research. 2013;15(4):e62 Epub 2013/04/19. 10.2196/jmir.2503 23594933 PMC3636321

[20] Lyles CR, Lopez A, Pasick R, Sarkar U. "5 mins of uncomfyness is better than dealing with cancer 4 a lifetime": an exploratory qualitative analysis of cervical and breast cancer screening dialogue on Twitter. Journal of cancer education: the official journal of the American Association for Cancer Education. 2013;28(1):127–33. Epub 2012/11/08. 10.1007/s13187-012-0432-2 . 23132231

[21] Myslin M, Zhu SH, Chapman W, Conway M. Using twitter to examine smoking behavior and perceptions of emerging tobacco products. Journal of medical Internet research. 2013;15(8):e174 10.2196/jmir.2534 23989137 PMC3758063

[22] Creswell JW, Creswell JW. Qualitative inquiry and research design: choosing among five approaches. 3rd ed Los Angeles: SAGE Publications; 2013 xxi, 448 p. p.

[23] Fishbain DA, Cutler RB, Rosomoff HL, Rosomoff RS. Validity of self-reported drug use in chronic pain patients. The Clinical journal of pain. 1999;15(3):184–91. . 10524471 10.1097/00002508-199909000-00005

[24] Becker WC, Fraenkel L, Edelman EJ, Holt SR, Glover J, Kerns RD, et al Instruments to assess patient-reported safety, efficacy, or misuse of current opioid therapy for chronic pain: a systematic review. Pain. 2013;154(6):905–16. 10.1016/j.pain.2013.02.031 23601625 PMC3683236

[25] Cunningham JA. Using Twitter to measure behavior patterns. Epidemiology. 2012;23(5):764–5. Epub 2012/08/09. 10.1097/EDE.0b013e3182625e5d . 22872115

[26] Paul MW BC. Dredze M. What Affects Patient (Dis)satisfaction? Analyzing Online Doctor Ratings with a Joint Topic-Sentiment Model. Association for the Advancement of Artificial Intelligence. 2013.

[27] Tobias E. Using Twitter and other social media platforms to provide situational awareness during an incident. Journal of business continuity & emergency planning. 2011;5(3):208–23. . 22130339

